# Early Clinical and Functional Outcome of Rotator Cuff Tendinopathy of the Shoulder Treated with Platelet Rich Plasma Injection

**DOI:** 10.5704/MOJ.2107.009

**Published:** 2021-07

**Authors:** AR Pritem, VT Abraham, R Krishnagopal

**Affiliations:** Department of Orthopaedics, Mahatma Gandhi Medical College and Research Institute Sri Balaji Vidyapeeth University, Pondicherry, India

**Keywords:** rotator cuff tendinitis, platelet rich plasma, functional outcome

## Abstract

**Introduction:**

Rotator cuff tendinitis has been treated using various methods including physiotherapy, steroid injections and recently platelet rich plasma (PRP). Most of these methods aim at giving symptomatic relief rather than addressing the pathology. There is no clear consensus over the benefit of using PRP for tendinitis. We decided to do a prospective clinical study to demonstrate the efficacy of PRP and study the functional outcome in the treatment of rotator cuff tendinopathy.

**Material and Methods:**

Patients with shoulder pain for more than three months not responding to NSAIDs or physiotherapy with a diagnosis of rotator cuff tendinitis, confirmed by MRI, were included in the study. Patients with rotator cuff tear or any other shoulder pathology were excluded. We included a total of 30 patients who received 5ml of landmark guided PRP injection in the subacromial space followed by a six-week exercise program. Patients were followed-up at 3, 6 and 12 weeks. and were assessed clinically using the VAS, SPADI and Constant and Murley Score.

**Results:**

VAS score of patients improved from a pre injection score of 7.4 to a score of 1.9 in the 12th week. The mean SPADI score and Constant score improved from a pre injection score of 73.33 and 39.57 to a post injection score of 18.1 and 86.47, respectively.

**Conclusion:**

Platelet Rich Plasma injections showed good to excellent early results, in patients with rotator cuff tendinopathy with improvement in VAS, SPADI and Constant scores.

## Introduction

Anatomical location of rotator cuff muscles and its biomechanics predisposes it to degenerative changes and because of this the rotator cuff is susceptible to tendinopathy, especially in the presence of risk factors like overuse, ageing, trauma, metabolic disorders or obesity^1^.

Lesions off the rotator cuff start of as acute tendinitis and may progress on to chronic tendinosis and then even partial tears of the rotator cuff if left untreated. Conservative treatment measures for cuff tendinopathies have given variable results, because most of these measures aim at giving symptomatic treatment rather than addressing the pathology^2^.

Traditional treatment methods include non-steroidal anti-inflammatory drugs, corticosteroid injections, ice, rest, and bracing or immobilisation and physiotherapy^3^.

Corticosteroid injections are uselful in only acute and subacute tendinits and some reports have said that they cause an inhibition in collagen synthesis which may lead on to tendon failure^4^.

The inadequacy of these therapies in some cases calls for new research. Research in the last decade has improved our knowledge about the tendon biology and pathology, resulting in the development of biological interventions for the disorders of the tendons.

The primary goal is to get pain relief and to increase the functional capacity of the involved tissue. In tendinitis there is a lack of inflammatory response as reported in histological studies resulting in poor tendon healing^5^. Therefore, treatment should not only target pain reduction, but also provide an environment where healing is stimulated, promoting tissue regeneration and ultimately pain relief without any recurrences.

Along with new insights regarding tendinopathies, a promising new era of biologically based cellular therapy has emerged. Orthobiologics involve the inclusion of biology and biochemistry in the development of bone and soft tissue replacement materials for skeletal and tissue healing^6^. Platelet Rich Plasma (PRP) therapy is the readily available autologous “bedside” injectable orthobiologic, which is gaining widespread use. It increases healing potential, mediates inflammatory processes and reduces pain through the release of mediating amines. PRP has become an important prophylactic alternative in pain medicine and in the treatment of chronic tendon pathology^7^.

Platelet-rich plasma is an autologous biomaterial obtained by centrifuging whole blood. PRP may be defined as a fraction of autologous plasma with platelet concentration above the baseline. Platelets are responsible for promotion of hemostasis, formation of new connective tissue and revascularisation. Thus peritendinous injection of PRP acts by limiting damage and promotes healing mechanisms in the tendons involved. It acts by its anti-inflammatory, anabolic and milieu interior altering mechanism through release of growth factors present in the platelets^8^.

Various studies have shown the positive effects of PRP in different clinical situations and it is a safe strategy to deliver the natural mixture of biologically active molecules within the tendon compartment to induce positive changes in the tendon microenvironment^9-11^. We decided to do a prospective clinical study to demonstrate the efficacy of PRP and to evaluate the functional outcome in the treatment of rotator cuff tendinopathies.

## Materials and Methods

Patients who presented to the outpatient clinic with shoulder pain and diagnosed as rotator cuff tendinopathy were enrolled in our study. Our inclusion criteria were patients more than 18 years of age; pain more than three months refractory to conservative treatment, rotator cuff tendinopathy as diagnosed by clinical examination and ultrasound/MRI. We excluded patients with concomitant rotator cuff tear, arthritis or bony lesions of shoulder, patients with inflammatory disease e.g., rheumatoid arthritis and patients who had local pathologies like skin lesions or infection.

The study was carried out after approval from our institutional ethics committee. The period of our study was from November 2015 to October 2017. We included a total of 30 patients who met our inclusion and exclusion criteria. From all patients a detailed clinical history of the patient was elicited. For all patients, a general physical examination was done, and the shoulder involved was examined. Tenderness if any, the range of motion, impingement signs, and rotator cuff power was recorded. Plain antero-posterior radiograph, axillary view radiographs and Ultrasound or MRI of the shoulder was taken in all patients. Patients were asked to fill the patient information sheet and were explained in detail about the study and informed written consent was obtained. The baseline SPADI (shoulder pain and disability index) score, Constant and Murley score and the VAS score were assessed and then patients were planned for intra-articular injection.

Under all aseptic precautions, about 20ml of blood was drawn from the antecubital vein of the patient and centrifuged to get 5ml to 6ml of platelet rich plasma. 1ml of the sample was sent to check for the platelet count to establish platelet concentration. The PRP without any buffering or activating agent was taken in a syringe. Using sterile aseptic precautions 5ml of prepared PRP was injected 1cm below and lateral to the posterolateral corner of the acromion into the subacromial space around the rotator cuff tendon. Sterile dressing was applied at the injection site. The patient lay supine for about 15 minutes without moving his shoulder. Patients were strictly observed for complaints such as pain, swelling and redness following injection. For pain ice pack application was given and patients who had severe pain were given either oral Acetaminophen or Tramadol. Patients were taught and told to do rotator cuff strengthening exercises from the 1st week onwards.

Patients were followed at 3rd, 6th, and 12th week. At each follow-up patient were assessed clinically for pain relief and range of motion. VAS score, SPADI score and Constant and Murley Score were recorded at each visit. Adverse reactions if any were noted.

The power analysis indicated a beta of 100%. Data was collected using a semi structured proforma. The data was analysed in the study using SPSS version 23. All the outcome variables had at least once a not normal distribution at some point in the timeline found by applying Shapiro Wilk test. A p value of less than 0.05 indicated not normal distribution. To compare the mean scores collected pre injection and post injection, Friedman’s test was employed due to non-parametric nature of data and presence of four related groups. A p value of <0.05 was considered as statistically significant.

## Results

**Fig. 1:**
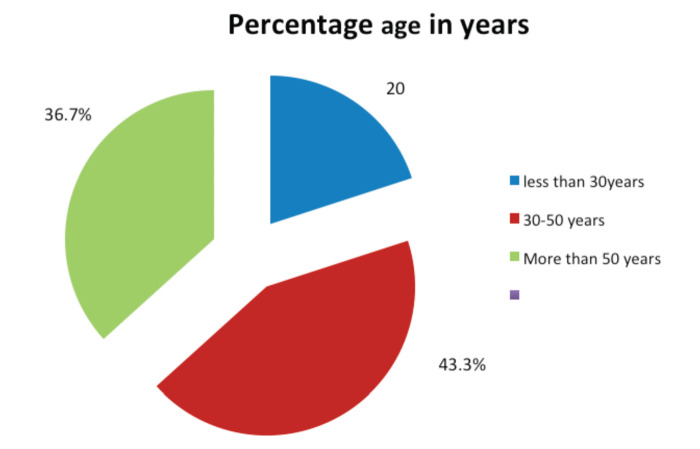
Age distribution in percentage.

A total number of 30 patients with rotator cuff tendinopathy were selected based on our inclusion and exclusion criteria. None of our patients were lost to follow-up. Majority of patients were between 30-50 years (43.3%), 36.7% of patients were more than 50 years and 20% of the patients were less than 30 years old (Fig. 1).

Majority of patients were males 17 (56.7%) and rest 13 (43.3%) of patients were females. Right shoulder was affected in 20 (66.7%) patients and left in 10 (33.3%).

The mean platelet count was 223.2±41.4×103⁄ μL in the whole blood samples and 994.9±210.3×103⁄ μL in the PRP samples. A 4-fold increase was seen in the number of platelets in PRP compared with whole blood.

The mean Visual Analog Scale score of patients improved from a pre injection score of 7.4±1.19 to a score of 1.9±1.09 in the 12th week. The magnitude of VAS had decreased significantly post injection with P value of less than 0.05 and with a strong effect size (Kendall’s W-0.997) (Table I).

**Table I:** Mean Visual Analog scale

Timeline	Visual analogue scale					
	Mean	SD	Kendall’s W	CHI square	d.f	P value
Pre injection	7.40	1.19	0.997	89.71	3	0.001
3rd week	5.43	1.19				
6th week	3.60	1.32				
12th week	1.90	1.09				

The pre injection mean SPADI score was 73.30 ± 9.02. Post injection SPADI at third, sixth and twelfth weeks were 55.76 ± 12.52, 38.56 ± 15.91 and 18.10 ± 10.65, respectively. The magnitude of SPADI had decreased significantly post injection with P value of less than 0.05 and with a strong effect size (Kendall’s W-0.987) (Table II).

**Table II:** Mean Shoulder Pain and Diability index (SPADI)

Timeline	SPADI					
	Mean	SD	Kendall’s W	CHI square	d.f	P value
Pre injection	73.33	9.02	0.987	88.84	3	0.001
3rd week	55.76	12.52				
6th week	38.56	15.91				
12th week	18.10	10.65				

The pre injection mean constant and Murley score was 39.57 ± 17.02. Post-injection mean, Constant, and Murley score at third, sixth and twelfth weeks were 56.80 ± 14.36, 70.17 ± 14.68 and 86.47 ± 8.59, respectively. The magnitude of Constant and Murley score had increased significantly post injection with P value of less than 0.05 and with a strong effect size (Kendall’s W-0.958) (Table III).

**Table III:** Mean Constant and Murley score

Timeline	Constant and Murley score					
	Mean	SD	Kendall’s W	CHI square	d.f	P value
Pre injection	39.57	17.02	0.958	86.17	3	0.001
3rd week	56.80	14.36				
6th week	70.17	14.68				
12th week	86.47	8.59				

**Fig. 2:**
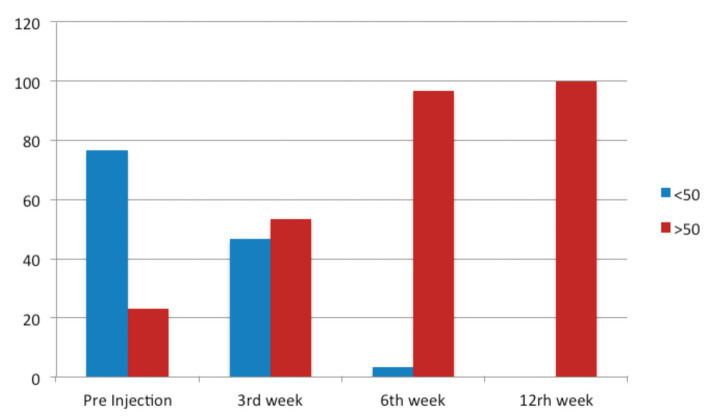
Improvement of shoulder pain and disability index (SPADI) with time (3rd, 6th, 12th week).

Over time the pre-injection VAS median of 7.5 had decreased to 6, 3.5, and 2 in 3rd, 6th, and 12th week, respectively (Table IV). Pre injection, all patients (100%) had a SPADI score more than 50 (Fig. 2). During the follow-up period, the pre injection SPADI median of 73 had decreased to 57, 37 and 15.5 in the 3rd, 6th, and 12th week post-injection, respectively (Table V).

**Table IV:** Median VAS over time

Timeline	Visual analogue scale				
	Min	25th	50th	75th	max
Pre injection	5.0	6.75	7.50	8.0	9.0
3rd week	3.0	4.0	6.0	6.0	7.0
6th week	2.0	2.0	3.5	5.0	6.0
12th week	1.0	1.0	2.0	2.25	5.0

**Table V:** Median SPADI over time

Timeline	SPADI				
	Min	25th	50th	75th	max
Pre injection	58.0	65.75	73.0	79.25	89.0
3rd week	21.0	49.0	57.0	63.0	78.0
6th week	6.0	26.5	37.0	50.0	85.0
12th week	3.0	10.0	15.5	24.25	43.0

**Fig. 3:**
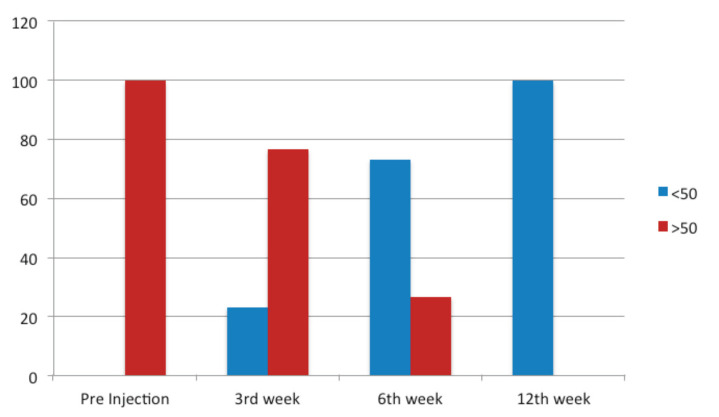
Improvement of Constant and Murley Score with time (3rd, 6th, 12th week).

Pre-injection, majority of the patients (76.7%) had a Constant Murley score less than 50 (Fig. 3). The pre injection median of Constant and Murley score was 36.5. The above median had increased post injection to 55.5, 69.5 and 90 during the 3rd, 6th, and 12th week follow-up, respectively, showing an increase in the score over time (Table VI).

**Table VI:** Median Constant and Murley score over time

Timeline	Constant and Murley score				
	Min	25th	50th	75th	max
Pre injection	16.0	25.5	36.5	50.5	72.0
3rd week	34.0	44.75	55.5	68.5	84.0
6th week	22.0	63.0	69.5	84.25	91.0
12th week	55.0	84.5	90.0	92.25	95.0

## Discussion

Rotator cuff tendinopathy has a multifactorial origin, which may include both intrinsic, and extrinsic factors. The intrinsic factors include degeneration of the cuff, which is age related and vascularity changes in the tendon substance. Extrinsic factors like biomechanical and anatomic dysfunctions, which can lead on to impingement, overuse, and overload of the tendons^12^. The treatment of tendinopathy is aimed at addressing these etiological factors.

PRP injections have been used over the past 10 years for chronic tendon injuries because of its properties that help in collagen synthesis, proliferation of tendon cells and improved vascularisation of the tendon^7,8^. We evaluated our results and compared them with those obtained by various other studies in literature.

In our study, rotator cuff tendinopathy was more common in the 4th and the 5th decade with a mean age of 43.3 years. The average age in our study was comparable to the studies of Kesikburun *et al*^12^, Mautner K *et al*^9^, Wesner *et al*^10^, Scarpone *et al*^11^ who had average age of 45.5 years, 48.6 years, 44.6 years, 46.2 years, respectively. Thus, rotator cuff tendinopathy is more common in the middle age, as compared to rotator cuff tears, which is more common in the elderly.

Our study had a male preponderance with 17 male and 13 female patients. Scarpone *et al*^11^ in their study of PRP for chronic tendinopathy had a higher male preponderance with 15 male and only 4 female patients. The higher male preponderance could be because males are more likely to be involved in heavy physical activities like manual labour, sports activities etc. Most of the males in our series of patient were either manual labourers or farmers.

Mautner *et al*^9^ did a retrospective study where they studied 180 patients. Platelet rich plasma injection for chronic tendinopathy was given in all these patients. In their study the mean VAS score improved from 7 to 2 at the end of 6 months, whereas in our study mean VAS score reduce from 7.4 to 1.9 at 12th week. They reported as moderate improvement in pain symptoms (>50%) with multiple PRP injection for chronic tendinopathy, our study had good results with single PRP injection. There was significant improvement in pain in most of our patients and they were able to get back to their activities of daily living.

Wesner *et al*^10^ did a randomised control trial on patients receiving PRP and reported clinical improvement in pain (>1.5/10 on VAS), disability score (>15 points DASH change), and tendon pathology while those receiving placebo injections did not. In the observational cohort, statistically and clinically significant improvements in pain and disability were observed.

Scarpone *et al*^11^ in their prospective open label study on 18 patients who received a single PRP injection, concluded that PRP resulted in safe, significant, and sustained improvement in pain and function. Twelve participants were “completely satisfied”, five patients were “satisfied”, and one participant was “unsatisfied”. The mean VAS score improved from 7.5 to 0.5 at 12th week. Our results were similar with mean VAS 7.4 to 1.9 at 12th week, as they had good improvement in VAS scores and majority of patients were satisfied.

Kesikburun *et al*^12^ did a comparative study of PRP versus Saline injection in 40 patients with rotator cuff tendinopathy. In their study the SPADI score, which was 77.5 reduced to 27.6 at the end of the 12th week in the patients who were treated with PRP. In our study the mean SPADI score reduced from 73.3 to 18.1 at the end of 12th week. We had better reduction in SPADI score. In their study mean VAS score reduction was from 8 to 3 at 12 weeks and in our study mean VAS score reduced from 7.4 to 1.9 at the 12th week. They concluded at one year follow-up, a PRP injection when compared to placebo was no more effective in improving the quality of life, pain, disability, and shoulder range of motion in patients with chronic rotator cuff tendinopathy who were treated with an exercise program.

Jane Fitzpatrick *et al*^13^ in their meta-analysis of randomised controlled clinical trials in which they included 18 studies, found that there is good evidence to support the use of a single injection of highly cellular leucocyte rich PRP under ultrasound guidance in tendinopathy. The preparation and the technique of intra tendinous injection technique of PRP have a clinical significance.

Rha *et al*^14^ in their randomised controlled trial compared dry needling vs PRP in patients with supraspinatus tendinosis in 39 patients. They reported that the platelet rich plasma injection leads to a reduction of pain and disability progressively, in comparison with dry needling. The mean SPADI index at 6 months was 17.7 ±3.7, for the PRP injection group and 29.5 ± 3.8 in the dry needling group (P<0.05). They concluded that PRP injections are safe and useful for rotator cuff disease. In our study the mean SPADI score was 18.1 at the 12th week.

We used only a single injection of PRP. Serial injections are one of the controversial topics in PRP injection. A second injection can be performed at one to two months if no therapeutic effect is seen, but in most of our patients a single injection was effective.

All our injections were landmark guided, given by a single surgeon trained in shoulder arthroscopy. Ultrasound guide may improve the accuracy of the injections, but the added cost was the reason we did not give the injections guided by ultrasound.

PRP is an evolving treatment modality gaining momentum in primary care, rehabilitation, and sports medicine applications. PRP may provide a minimally invasive outpatient treatment for refractory rotator cuff tendinopathy thus providing a less expensive alternative modality to surgery with a reduced potential for operative side effects and adverse events. Physiotherapy is an important part of the treatment, post PRP injection. Further studies with a larger number of patients will prove the efficacy of PRP in rotator cuff tendinopathy. Our study fortunately had no complication like needle breakage, local inflammation, or infection, except for the pain at the injection site for 24 to 48 hours.

The limitations of our study were that we had a smaller sample size and no controls, we did not do a post injection ultrasound or MRI to evaluate the tissue regeneration and healing. This may be, carried out in subsequent research efforts. Future studies with a longer follow-up are being planned.

## Conclusion

Platelet Rich Plasma injections showed good to excellent early results, in patient with rotator cuff tendinopathy with improvement in VAS, SPADI and Constant scores. It can be recommended for patient with rotator cuff tendinopathy not responding to physiotherapy.
